# Spiritual over physical formidability determines willingness to fight and sacrifice through loyalty in cross-cultural populations

**DOI:** 10.1073/pnas.2113076119

**Published:** 2022-02-07

**Authors:** Chad C. Tossell, Angel Gómez, Ewart J. de Visser, Alexandra Vázquez, Bianca T. Donadio, Amanda Metcalfe, Charles Rogan, Richard Davis, Scott Atran

**Affiliations:** ^a^Department of Behavioral Sciences and Leadership, Warfighter Effectiveness Research Center, United States Air Force Academy, CO 80840;; ^b^Center for Conflict Studies and Field Research, Artis International, Saint Michaels, MD 21663;; ^c^Departamento de Psicología Social y de las Organizaciones, Universidad Nacional de Educación a Distancia, 28040 Madrid, Spain;; ^d^Changing Character of War Centre, University of Oxford, Oxford OX1 3TD, United Kingdom;; ^e^Gerald Ford School of Public Policy, University of Michigan, Ann Arbor, MI 48109

**Keywords:** spiritual formidability, physical formidability, will to fight, self-sacrifice, loyalty

## Abstract

Across 11 studies involving six countries from four continents (*n* = 3,285), we extend insights from field investigations in conflict zones to offline and online surveys to show that personal spiritual formidability—the conviction and immaterial resources (values, strengths of beliefs, character) of a person to fight—is positively associated with the will to fight and sacrifice for others. The physical formidability of groups in conflict has long been promoted as the primary factor in human decisions to fight or flee in times of conflict. Here, studies in Spain, Iraq, Lebanon, Palestine, and Morocco reveal that personal spiritual formidability, a construct distinct from religiosity, is more strongly associated with the willingness to fight and make costly self-sacrifices for the group than physical formidability. A follow-on study among cadets of the US Air Force Academy further indicates that this effect is mediated by a stronger loyalty to the group, a finding replicated in a separate study with a European sample. The results demonstrate that personal spiritual formidability is a primary determinant of the will to fight across cultures, and this individual-level factor, propelled by loyal bonds made with others, disposes citizens and combatants to fight at great personal risk.

## Significance

Despite intermittent interest in and evidence of the importance of nonmaterial factors in war and other extreme forms of intergroup conflict, material factors such as optimal use of physical strength, manpower, and firepower remain the dominant concerns of US and allied military training, decision-making, and related academic literature. In this work, we demonstrate the cross-cultural primacy of personal spiritual over physical formidability on the will to fight in populations from the Middle East, Europe, and North America, including US cadets in whom stronger group loyalty mediates the effect. This empirical examination of spiritual formidability and its link between self and group in willingness to self-sacrifice aims to extend understanding of interpersonal and intergroup conflict and inform considerations of policy.

What provokes humans to fight and sustain the suffering costs of war? In 2021, this question became pivotal following the crushing defeat of a vastly more materially endowed military—the US-backed Afghan National Defense Security Forces—at the hands of the Taliban, a force initially much smaller and persistently inferior technologically. The Taliban’s victory was shocking to many allied political and military planners, as the rout of tens of thousands of US-trained and equipped Afghan forces, following the US decision to withdraw its military, was both stunningly rapid and complete. Taliban fighters endured America’s longest war, sustaining a conflict for two decades despite heavy casualties and their opponents’ significant investments in establishing and defending a democratic Afghanistan. US President Joe Biden acknowledged the failure of material advantage in this seemingly surprising outcome: “We spent over a trillion dollars. We trained and equipped an Afghan military force…incredibly well equipped—larger in size than the militaries of many of our NATO allies—what we could not provide them was the will to fight” (1).

The will to fight—an individual’s willingness to mobilize and seek success in violent conflict regardless of the risk of death or injury (2)—has been studied by social scientists for decades. Existing research has shown perceptions of relative physical formidability at individual and group levels inspire humans to fight, flee, or negotiate across evolutionary history (3, 4). Physical formidability, defined as the material capacity to inflict damage on an opponent, has been proxied at the individual level by body size and musculature, self-perceived physical strength, weightlifting ability, and predicted aggression and endorsement of coalitional aggression including interstate war (5). As self-perceived physical strength increases, humans are more willing to engage in violent interpersonal confrontations (6). Physically stronger individuals are perceived to have a higher likelihood of surviving combat and inflicting harm on others (7–9). Individual physical formidability is magnified when, joining others, the group as a whole as well as each of its members seem more physically formidable (8). Group physical formidability, which has been assessed by ingroup size, strength, and cohesiveness and—in operational terms, manpower and firepower—contributes to perceptions of relative formidability leading to decisions to fight sometimes removed from calculations of personal risk (5).

In certain real-world contexts, however, including life-and-death situations, we suspect that self-perceived spiritual formidability, relative to self-perceived physical formidability, is more influential toward inspiring a willingness to fight and sacrifice. The importance of spiritual formidability—the conviction and nonmaterial resources (values, strength of beliefs, and character) of a person or a group to fight and achieve their goals in conflict—was identified as a critical component of esprit de corps in recent ethnographic and psychological research among frontline fighters (10). At the group level, their esprit provided the inspiration to fight despite significant risk of death. The religious fighters of the Islamic State of Iraq and the Levant (ISIS) and secular Marxist–Leninist fighters of the Kurdistan Workers' Party (PKK) spontaneously opposed the importance of physical formidability over spiritual formidability (or ruhi bi ghiyrat in both Arabic and Kurdish) to defend what is most cherished. When asked to further characterize spiritual formidability, they routinely described it as “strength of belief in what we are fighting for” and “what is in our heart” (10). These sentiments are evidently personal. As recent studies suggest that perceptions of formidability involve more than simple comparisons of physical size and strength (11, 12), we anticipate that an individual’s self-perceived spiritual formidability can play a critical role toward inspiring the will to fight and sustain conflict.

In addition, just as self-perceived physical formidability is magnified when combined in groups, we suspect a similar mechanism is at work connecting individual-level (personal) spiritual formidability to the willingness to engage in conflict through loyalty to an ingroup. Loyalty is multifaceted and characterized as intensely spiritual (13), familial (14), and functional (15). In conflict, loyalty refers to the allegiances developed and felt among teammates (16), ingroup superiors (17), and a primary group (18). The attachments established through loyalty toward others within a primary group have been found to be instrumental in decisions to commit violence against others, including civilians (16, 19–21). In previous work, intergroup conflict is driven by the need to defend members of the ingroup (20, 21). Our aim in the current work is to extend this research by assessing loyalty as a mediator between personal spiritual formidability and the will to fight and sacrifice for others. Specifically, we hypothesize that personal spiritual formidability by itself represents the personal strength of an individual to fight an opponent. Through loyalty to an important ingroup, defending others in conflict becomes an even greater motivating force.

Personal spiritual formidability, then, may represent a primary source of strength and group loyalty a related means of protecting the group against enemies. We examine this hypothesis here through a series of 11 studies. In the initial three studies, we explore the relationship between self-perceived physical formidability and self-perceived spiritual formidability. Because of the potential conflation of personal spiritual formidability with religiosity, we also examine these constructs to understand their differences. Next, we assess the relative influence of personal spiritual formidability vis-à-vis physical formidability on the will to fight among participants in six studies. Finally, we test our hypothesis that loyalty, as a collective orientation, mediates the influence of self-perceived spiritual formidability on the will to fight in two studies. The replicability and external validity of our predictions are assessed among populations from different cultures and countries, inside and outside conflict zones, using insights from field studies to inform both offline and online surveys in Iraq, Morocco, Palestine, Lebanon, and Spain. To reinforce the ecological validity of our findings, we examine the effect for those who have decided to fight and die for others and realistically have the opportunity to sacrifice in the line of duty, namely, cadets of the US Air Force Academy (USAFA).

## The Nature of Spiritual Formidability and Its Relationship to Religiosity.

To date, much of the speculation and research relating spirituality to conflict has focused on religion. Followers of major religions arguably have maintained an advantage through ingroup connections that are stronger than cooperation experienced in other groups. The extreme solidarity, practices, and values of many religions elicit a sense of commitment that has galvanized men and women for war and a willingness to fight and die. For example, ritual attendance predicts support for the willingness to fight and die in Palestine, Israel, India, Mexico, Russia, and other populations (22) and positively correlates with the will to fight in times of war (23). Perceived supernatural support increases willingness to engage in violence and battle confidence (24, 25). When reminded of God, people can be more willing to take risks despite personal harm (26), better perceive the social support of comrades, and experience reduced anxiety in risky situations (27), including less fear of death (28).

Although spiritual formidability emphasizes convictions in values, beliefs, and ideologies, it is not simply the strength of religious conviction. Spirituality in philosophy (29) and psychology (30) is often characterized more broadly as relating to notions of transcendental truths and means in the search for significance (cognitive, see ref. 31), connectedness to others (emotional/social, see ref. 32), to Nature or Providence, and commitment to a higher set of principles that guides daily living and personal and group morality and behaviors (33). Whether expressed through religion or not, spirituality as a construct has garnered a renewed interest in a wide range of fields, including education (34) and character development (35). Although research on spirituality applied to combat is limited and primarily focused on religion and ties to mental health, studies on spirituality provide plausible evidence of the connection between personal spiritual strength and the will to fight. Spirituality seems to provide frontline combatants with emotional comfort and a sense of justified effort amid harsh conditions, intense combat experiences, and extreme personal risk (36). In a large survey of 1,250 soldiers in a combat zone, spirituality moderately correlated with moral courage, moral efficacy, and resilience (37). Because of these limited findings, we suspect that spiritual formidability, as a construct tying religious or nonreligious spirituality to conflict, can drive individuals to fight and sacrifice. Still, spiritual formidability may overlap with religiosity; thus, our first three studies examine whether religiosity and spiritual formidability are distinct or related constructs and what their relationship is with self-perceived physical formidability.

## Spiritual and Physical Formidability.

In conflict, relative formidability is quickly summarized by individuals taking into account both physical and psychological dimensions (i.e., the Formidability Representation Hypothesis) (38). Physical formidability, as the material capacity to inflict damage on an opponent, has arguably provided an adaptive advantage in conflict throughout human history (38, 39). As one’s physical size, strength, and access to resources increase, so does estimated physical formidability (39). Bigger and stronger individuals tend to be more aggressive and have a reduced risk of harm to themselves when engaged in conflict (5, 9). In large modern samples, self-reported physical strength predicted intentions to participate in political violence (5). The advantages provided by relative physical formidability are both ancient and recurrent throughout history and supported by a large body of empirical research (3–9, 11, 38–41).

Testable characterizations of formidability, although generally conceptualized and measured by physical features (e.g., height, weightlifting, and access to weapons), have recently been expanded to include the nonphysical, psychological factors contributing to an individual’s summary representation of relative formidability between friend and foe (11, 12, 24). For example, in one recent study (24), perceived supernatural support enhanced self-assurance in a knife fight. This line of research suggests that an individual’s perceived physical formidability combines a myriad of physiological, psychosocial, and sociotechnical factors into a single concept that could contribute to decisions in violent contexts. As Scriver and colleagues note (12), “Formidability is the product of physical, social, technological, and psychological factors—a strong fighter dominates a weak fighter; a fighter with many allies dominates a lone antagonist; a well-armed individual dominates a poorly armed opponent; and, critically, an aggressive, motivated fighter dominates a meeker or less motivated foe.” As such, the contributions of nonmaterial factors in assessments of formidability and the will to fight, contrasted with material factors, can shed light on the relative importance of these factors in battlefield decision-making.

Accordingly, by strictly characterizing spiritual formidability in our current studies as a construct distinct from, yet possibly related to, physical formidability, we may find that the physical dimension is not sufficient, or even paramount, in certain life-and-death decision-making scenarios. Nonmaterial factors may not be simply involved in the decision calculus, but predominant. We examine personal spiritual formidability, then, as an explicitly nonmaterial factor representing the nonphysical resources (conviction, bravery, internal energy, values, strength of beliefs, and character) comprising the capacity (inner strength) of a person to achieve their goals in conflict. In these studies, we suggest that individual spiritual formidability is a basic predisposition to inspire the will to fight and sacrifice and may be primary in the summary representation of formidability toward decisions to fight or flee.

This hypothesis is consistent with other literature suggesting that nonmaterial factors such as ideological commitment and resolve can help mobilize forces and yield greater effectiveness on the battlefield. For example, in US and allied military circles, recent reports suggest that material factors alone cannot account for critical historical outcomes, including the French resistance at Verdun in World War I, Soviet persistence against the initially overwhelming German onslaught in World War II, Communist Vietnam’s resistance and eventual victory against French then American military might, Afghan resistance to the Soviets and Americans, and ISIS’s defeat of the Iraqi army (42). In interviews with combatants, esprit was noted as the single most important influence for inspiring youth to fight in combat (43). Two decades of war culminating in the 2021 events in Afghanistan appear to forcefully raise the possibility that the spiritually determined Taliban outlasted and outbattled physically superior US, allied, and Afghan national military forces.

For these reasons, we explore the relative contributions of nonmaterial factors to assessments of relative formidability and the will to fight and contrast these with material factors. We suspect self-perceived spiritual formidability to be more important than self-perceived physical formidability, and we test this in Studies 4 through 9. The spiritual dimension in warfare has broadly been underestimated because theories such as Lanchester’s Laws of Combat (which uses mathematical modeling to characterize fighting strength and war outcomes as proportional to group size and potency—see ref. 44) downplay the role of beliefs, values, and ideology in favor of material might as primary determinants of wartime decisions and outcomes. Although it is plausible that physical formidability relates to expectancy of victory in conflict and influences the will to sustain the hardships of conflict, previous research suggests that spiritual formidability, grounded in value and convictions, could have a greater effect in some real-world contexts for individual-level engagement in conflict.

## Connecting Personal Spiritual Formidability to the Will to Fight through Loyalty

Although self-perceived spiritual formidability can provide citizens the necessary motivation to mobilize and fight, we suspect this is explained through loyalty to others. We examine the proposition that self-perceived spiritual formidability provides the personal disposition to inspire concerted collective action through loyalty. Loyalty has been described and analyzed as a cognitive activity, a moral emotion, a guiding virtue (45), a contract between two people, and as a set of prosocial behaviors on behalf of the group (16). Under combat conditions, loyalty can be felt as intensely spiritual (13), driving a connection to teammates to become more familial than real kin (14, 19). Military groups tend to deliberately cultivate a special type of ingroup attachment that encourages loyalty between group members (16). This type of loyalty among combatants may provide sustained motivation to initiate and suffer violence. Indeed, loyalty to comrades in arms has led to violence on the battlefield beyond what seems rational and at high personal risk (14, 46). Militaries therefore deliberately inculcate this special type of ingroup attachment to incite greater loyalty to group members and discourage empathy toward the outgroup (16). Insofar as loyalty is an inner virtue linking the self with the group, we expect that loyalty will connect self-perceived spiritual strength with fighting and sacrificing for important others.

This was evidenced in a previous study (10) in which we demonstrated that group-level spiritual formidability was vital to motivating extremists to commit violence. A large majority of participants in that work referred to spiritual formidability as deeply personal in terms of convictions (strength of beliefs and values) and internal strength (“character in pursuit of goals and facing adversities,” “heart,” and “energy”). Because their identities were thoroughly fused with their ingroup, their dispositions to fight were firmly entrenched in the ingroup (47–49). The group’s spirit (esprit de corps) promoted the shared pride and belonging to a unit inspiring enthusiasm, devotion, and strong regard for the honor of the group. Members of ISIS and the PKK rejected notions of physical strength as vital to their motivation in conflict and explicitly noted the primacy of spiritual strength in connecting them to their group. As such, the strong bonds they forged in conflict recalled the spiritual ties that World War II veterans reported experiencing in combat (43) and what early theorists, such as Sun Tzu, noted as vital to success:

The art of war is of vital importance to the State. It is a matter of life and death, a road to safety or ruin. Hence, it is a subject of inquiry which can on no account be neglected… and he will win whose army is animated by the same ***spirit*** throughout all its ranks. (Sun Tzu, *The Art of War*) (50) 

For ISIS and PKK fighters, the interplay between individual-level and group-level spiritual formidability was so strong that it was not possible to disentangle (at least with theories and measures available to us). Their sentiments were simultaneously personal and connected. They conveyed the importance of the deep-seated beliefs, values, and ideologies reminiscent of the pursuit of a personal “sense of significance” and that is recurrently associated with the willingness to engage in radical behaviors and extreme violence (31). This arguably inspired them to action at high personal risk in connection with others. Thus, we suspect personal spiritual formidability to be the basic determinant of the will to fight at an individual level that supplies citizens with the core strength to fight and potentially die for others. As these “others” become less imagined and more real by way of being part of a group, loyalty mediates the willingness to fight. We examine this hypothesis through a mediation analysis in Studies 10 to 11.

In sum, nonmaterial factors often have been relegated to secondary or insignificant factors in the motivational makeup of combatants. Although there is sporadic, mostly anecdotal evidence that nonmaterial personal convictions may be significant factors in combat and other forms of intergroup violence, there is a lack of replicable empirical research that systematically explores this link and mechanisms to connect the individual to the group in different cultural settings. This series of investigations aims to help fill this gap.

## Overview of 11 Studies and Hypotheses

To this end, we conducted 11 studies from populations in six countries to test our hypotheses that a) spiritual formidability is distinct from religiosity, b) self-perceived spiritual formidability is related to self-perceived physical formidability, c) the former (spiritual formidability) is more strongly related to the expressed willingness to sacrifice relative to physical formidability, d) this positive association is generalizable across different samples, and e) the psychosocial factor connecting spiritual formidability to willingness to fight and sacrifice is loyalty to the group. We began with three online studies in Spain to understand spiritual formidability as a construct and how it relates to religiosity. Then, using a mix of data collection strategies, we present six studies comparing spiritual formidability with physical formidability in six countries with populations of different natures [e.g., Iraqi citizens living in Mosul after the expulsion of ISIS; Muslims living in urban Moroccan neighborhoods associated with previous terrorist bombing campaigns (51, 52)]. After examining the relationship between spiritual formidability and costly sacrifices, we then test the link among a sample of participants for whom the willingness to self-sacrifice is part of their ethos and expected behavior (USAFA cadets in Study 10). Finally, in order to generalize these findings, we test whether the mediating role that group loyalty plays in relating spiritual formidability to costly sacrifices for the group is replicated in a larger and different cultural sample (Spanish civilians in Study 11).

**Fig. 1.**
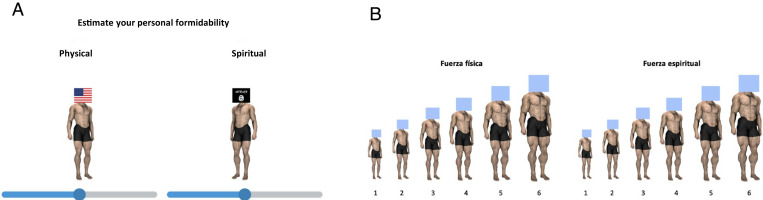
Measures for personal spiritual and physical formidability items presented on a (*A*) tablet and (*B*) paper-and-pencil survey for Study 7. Reprinted with permission from ref. 63.

To broaden and deepen the construct of formidability, we operationalize self-perceived personal spiritual formidability strictly as conviction and immaterial resources (values, strength of beliefs, and character) of a person or a group to fight and achieve their goals. We use the same visual measures of relative size and strength for both physical and spiritual formidability, distinguishing them only by different verbal and written frames. As Fessler, Holbrook, and colleagues note in several papers (3, 4, 6, 11, 12, 38, 39), the use of physical size and physical strength as measures of relative formidability among conflicting parties does not, and is not intended to, reflect only material assets and liabilities. Rather, owing to its phylogenetic antiquity and ontogenetic ubiquity, the dimensions of physical size and physical strength are used by a panhuman representational system to summarize any and all of the factors—material or nonmaterial—that constitute tactical assets or liabilities for each party in a potential conflict. In line with the significant body of evidence that these authors provide, we combine physical aspects of size and strength in a single scale that represents the minds-eye image of an opponent ranging from large and strong to small and weak (Fig. 1). This scale does not only reflect a person’s or group’s material assets but, under distinct verbal framings, can summarize and distinguish other aspects of formidability, including spiritual strength.

### Studies 1 to 3: Spiritual Formidability, Physical Formidability, and Religiosity.

Our first three studies in Spain were conducted online to understand self-perceived spiritual formidability vis-à-vis self-perceived physical formidability and religiosity. Study 1 (*n* = 202), using measures and procedures adopted from Fessler and colleagues’ seminal work (38), asked participants to respond to a questionnaire that included items measuring religiosity (*SI Appendix*) and perceived personal physical and spiritual formidability (Fig. 1). Participants increased or decreased the size and muscularity of an image of a male body corresponding to their perceptions of formidability. A small, thin figure corresponded to a value of zero, and the largest, most muscular figure corresponded to a value of one. Study 2 (*n* = 176) was designed to ensure the findings of Study 1 hold among a sample of individuals who consider themselves religious. Participants in Study 2 reported their religious practice level by selecting an option among three that best described their religious identity and level of practice (religious and practicing, religious but not practicing, neither). Like Study 1, we hypothesized spiritual formidability and religiosity to be orthogonal for participants. In Study 3 (*n* = 363), we asked participants what is more important to predict behavior: spiritual formidability or religiosity. We expected participants to distinguish religiosity and spiritual formidability and for perceptions of the latter to be more influential in predicting behavior.

Across all three studies, our hypotheses were supported. Spiritual and physical formidability were correlated, though weakly (Study 1: *r* = 0.21, *P* < 0.01 and Study 2: *r* = 0.19, *P* < 0.05). Self-perceived spiritual formidability was not correlated with religiosity in Study 1 (*r* = 0.10, *P* = 0.15) and Study 2 (*r* = 0.10, *P* = 0.20). Regardless of reported religiosity level, there was no relationship between religiosity and physical formidability (*P* > 0.52). Participants perceived their own spiritual formidability (Study 1: M = 0.69, SD = 0.24; Study 2: M = 0.71, 0.22) to be significantly greater than their personal physical formidability (Study 1: M = 0.51, SD = 0.25, *t* = 8.46, *P* < 0.001; Study 2: M = 0.49, SD = 0.22, *t* = 9.93, *P* < 0.001). The percentage of participants that consider spiritual formidability as more important than religiosity in predicting behavior (79.3%) was significantly higher (Χ^2^ = 124.98, *P* < 0.001). Across these three studies, spiritual and physical formidability appear to be distinct yet related, warranting further study (continued in Studies 4 through 11). Spiritual formidability is distinct from religiosity for both religious and nonreligious individuals, and the former is more influential in motivating behavior.

### Studies 4 through 9: Self-Perceived Spiritual and Physical Formidability on the Will to Fight.

Because of our interest in peoples’ willingness to fight and sacrifice, we conducted ethnographic field work among 1) young displaced males in in Mosul, Iraq, just after the defeat of ISIS (where the very concept of spiritual formidability first emerged, when both ISIS and PKK fighters spontaneously brought the notion to our attention, see refs. 13 and 14); 2) the general population from neighborhoods linked to previous terrorist campaigns (e.g., Sidi Moumen) in Casablanca, Morocco; 3) Palestinians across Gaza and the West Bank; and 4) Shia, Sunni, and Christian communities in Lebanon. We followed up in these populations with field surveys in and around Mosul (Study 4, *n* = 72) and in Casablanca (Study 5, *n* = 420), both offline and online studies across the West Bank in Palestine (Studies 6 to 8, full *n* = 1,371), and an online study in Lebanon (after the COVID outbreak, Study 9, *n* = 321). In each study, measures of spiritual and physical formidability were estimated as measured in Studies 1 to 3 (except, in Study 7, paper-and-pencil surveys were used, and formidability ratings were on a scale from 0 to 10). As detailed in *SI Appendix*, four- to six-item scales regarding their willingness to make costly sacrifices were used in each of the six studies (*α* values ranged from 0.88 to 0.95). We suspected perceptions of spiritual formidability to be related to physical formidability, though weakly, given the results from Studies 1 to 3 and previous work identifying a single representation of formidability (3). Additionally, we expected self-assessments of spiritual formidability to be greater than physical formidability regarding differences between means and their predictive power for the will to fight and sacrifice.

**Fig. 2.**
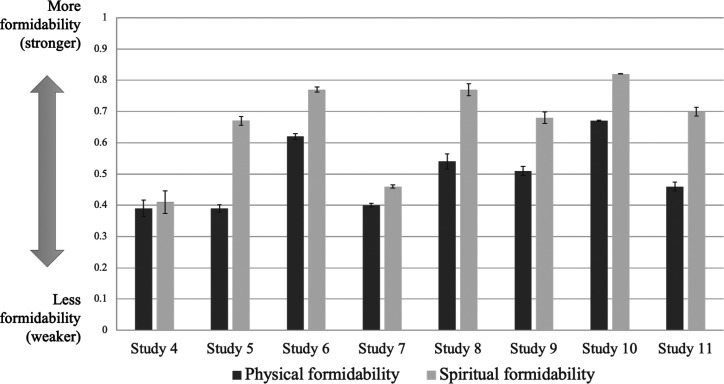
Comparison between perceived physical and spiritual formidability in Studies 4–11. Differences between these means were significant for Studies 5–11 (*P* < 0.001). Note: In Studies 7 and 10, physical and spiritual formidability were rescaled to make them comparable to the rest of the studies.

Table 1 includes the means, SDs, and correlations of physical formidability, spiritual formidability, and expressed willingness to make costly sacrifices for the ingroup. As theorized, spiritual formidability was positively associated with physical formidability in all six studies, though faintly, similar to Studies 1 to 3 supporting a unified summary representation of formidability. Participants in five of the six samples perceived themselves significantly stronger in spiritual relative to physical formidability supporting different mechanisms for physical and spiritual formidability (Fig. 2). The results from Study 4 yielded no real differences in mean ratings of spiritual versus physical formidability. At first blush, the differences between the results in Study 4 and the others could be attributed to gender given that the Iraqi sample was the only all-male sample (thus, potentially leading to increased perceptions of personal physical formidability). Moreover, unlike our previous study with frontline combatants in Iraq, in which perceived group spiritual formidability was a critical determinant of will to fight (and of actual battlefield casualty rates), our post-ISIS population pool consisted of displaced noncombatants (or defeated combatants). In fact, participants in Study 4 yielded the lowest self-perceptions across samples of physical formidability (M = 0.39) as well as spiritual formidability (M = 0.41).

**Table 1.** Means, SDs, and correlations for Studies 4 to 9

	M	SD	Physical	Spiritual	Sacrifices
Study 4 (Iraq, *n* = 72)					
Physical formidability	0.39_a_	0.23	—		
Spiritual formidability	0.41_a_	0.31	0.26*	—	
Costly sacrifices	2.57	1.74	0.01	0.23*	—
Study 5 (Morocco, *n* = 420)					
Physical formidability	0.39_b_	0.26	—		
Spiritual formidability	0.67_a_	0.29	−0.20***	—	
Costly sacrifices	1.66	1.49	−0.02	0.20***	—
Study 6 (Palestine, *n* = 730)					
Physical formidability	0.62_b_	0.25	—		
Spiritual formidability	0.77_a_	0.21	0.32***	—	
Costly sacrifices	3.93	1.77	0.11**	0.22***	—
Study 7 (Palestine, *n* = 470)					
Physical formidability	4.03_b_	1.34	—		
Spiritual formidability	4.60_a_	1.18	0.45***	—	
Costly sacrifices	3.96	1.73	0.21***	0.40***	—
Study 8 (Palestine, *n* = 171)					
Physical formidability	0.54_b_	0.32	—		
Spiritual formidability	0.77_a_	0.24	0.46***	—	
Costly sacrifices	3.90	2.24	−0.02	0.21*	—
Study 9 (Lebanon, *n* = 321)					
Physical formidability	0.51_b_	0.27	—		
Spiritual formidability	0.68_a_	0.32	0.30***	—	
Costly sacrifices	3.06	2.29	0.13*	0.34***	—

*P < 0.05; **P < 0.01; ***P < 0.001. Columns with different subscripts for physical and spiritual formidability differ at *P* < 0.001.

In all six studies, however, spiritual formidability significantly and positively correlated with costly sacrifices. Physical formidability correlated with costly sacrifices in Studies 6, 7, and 9, although to a lesser extent than spiritual formidability (Study 6, *z* = 2.05 *P* = 0.020; Study 7, *z* = 3.19, *P* = 0.001; and Study 9, *z* = 2.81, *P* = 0.002). A series of regressions of self-perceived physical and spiritual formidability on costly sacrifices show that only spiritual—not physical—formidability predicted costly sacrifices for the group (Table 2). The relationship between physical formidability and costly sacrifices in half of the studies follows previous research indicating assessments of physical formidability are at play in making decisions to sacrifice for others. Yet, the role of physical formidability in these predictions seems less uniform and significantly weaker than the influence of spiritual formidability. Across all six studies, the regression analyses reveal that spiritual formidability predicts costly sacrifices for the group, independent of the country, the sociopolitical setting of the sample, and the method of data collection.

**Table 2.** Regression analysis of personal physical and spiritual formidability on willingness to fight and commit costly sacrifices for their group (Studies 4 to 9)

Study	Predictor	*B*	*SE*	*t*	*P*	LLCI	ULCI
4	Physical formidability	−0.49	0.94	−0.52	0.605	−2.368	1.390
	Spiritual formidability	1.42	0.70	2.04	0.045	0.031	2.814
5	Physical formidability	0.11	0.29	0.38	0.703	−0.459	0.680
	Spiritual formidability	1.04	0.26	3.99	<0.001	0.527	1.553
6	Physical formidability	0.30	0.29	1.04	0.298	−0.265	0.864
	Spiritual formidability	1.76	0.34	5.16	<0.001	1.086	2.423
7	Physical formidability	0.04	0.06	0.67	0.505	−0.078	0.158
	Spiritual formidability	0.57	0.07	8.19	<0.001	0.429	0.701
8	Physical formidability	−0.97	0.65	−1.50	0.135	−2.252	0.308
	Spiritual formidability	2.59	0.85	3.03	0.003	0.898	4.272
9	Physical formidability	0.25	0.47	0.53	0.597	−0.671	1.165
	Spiritual formidability	2.33	0.39	5.96	<0.001	1.564	3.105

LLCI, lower-level CI; ULCI, upper-level CI.

### Studies 10 to 11: Loyalty as a Mediator of Spiritual Formidability and Will to Fight.

The consistency of findings across all nine studies supported our hypothesis that personal spiritual formidability is related to physical formidability and to costly sacrifices and has a stronger positive association with costly sacrifice relative to physical formidability across samples. To further assess the underlying mechanism of spiritual formidability in predicting costly sacrifices, we assessed cadets at the USAFA (*n* = 120) as well as a larger group from the general population in Spain (*n* = 240). We anticipated that 1) self-perceived spiritual formidability is positively related to loyalty and costly sacrifices, 2) loyalty is positively related to costly sacrifices, and 3) the positive relation between self-perceived spiritual formidability and costly sacrifices is mediated by loyalty. We expected loyalty, given its spiritual and practical dimensions (19), to be related to formidability perceptions and an important link toward inspiring the will to fight. As in Studies 4 through 9, participants were asked to estimate their personal physical and spiritual formidability, group loyalty, and the costly sacrifices they would be willing to make for their country (Study 10 α = 0.93, Study 11 α = 0.88). We examined the correlation between variables and the regression as before. Finally, we examined the extent to which loyalty to their ingroups mediated the positive relation between spiritual formidability and costly sacrifices, controlling for physical formidability, age, and gender.

**Fig. 3.**
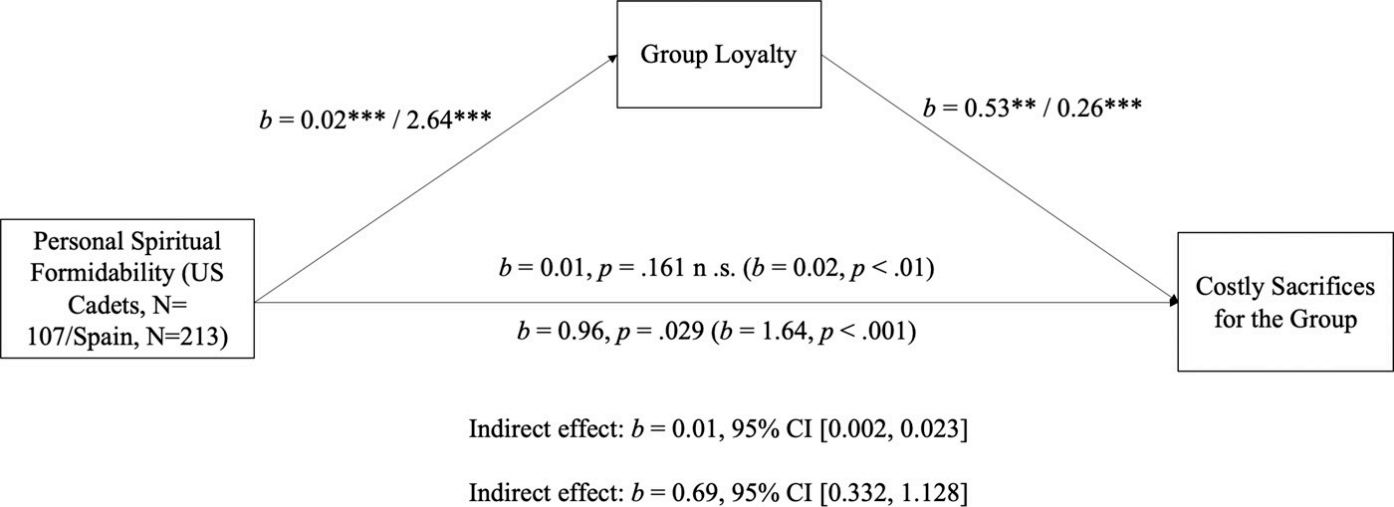
Mediation analysis shows that loyalty mediates the relationship between personal spiritual formidability and costly sacrifices for the group. Note: The sample sizes reported here for Study 10 (US cadets) and Study 11 (Spain) do not exactly match the originally reported sample sizes. This is because there were some missing cases in both studies owing to participants not responding to all items. Review boards for our studies mandated that some response items were optional for participants.

Table 3 includes the means, SDs, and correlations of physical formidability, spiritual formidability, and costly sacrifices for the group. As in Studies 1–9, cadets and Spanish civilians perceived themselves significantly stronger in spiritual than physical formidability (Fig. 2). Spiritual formidability significantly and positively correlated with physical formidability and costly sacrifices for the group. Table 4 shows the result of regressing physical and spiritual formidability on costly sacrifices. For cadets, only spiritual, not physical, formidability predicted costly sacrifices. Spanish civilians yielded remarkably similar results. Participants perceived themselves significantly stronger in spiritual than in physical formidability. Spiritual formidability significantly and positively correlated with costly sacrifices for the group, loyalty to the group, and loyalty to other people in general. Group loyalty correlated with costly sacrifices for the group. Again, in this sample, spiritual, not physical, formidability through loyalty to the group predicted costly sacrifices (Fig. 3).

**Table 3.** Means, SDs, and correlations for Studies 10 to 11

	M	SD	Physical	Spiritual	Sacrifices
Study 10 (US cadets, *n* = 120)					
Physical formidability	67.72_b_	21.08	—		
Spiritual formidability	82.37_a_	15.00	0.26**	—	
Costly sacrifices	5.76	1.41	−0.01	0.24*	—
Loyalty	6.53	0.77	0.05	0.31**	0.33**
Study 11 (Spain, *n* = 240)					
Physical formidability	0.46_b_	0.20	—		
Spiritual formidability	0.70_a_	0.20	0.15*	—	
Costly sacrifices	0.90	1.32	0.14*	0.27***	—
Loyalty	3.54	1.88	0.19**	0.34***	0.43***

*P < 0.05; **P < 0.01; ***P < 0.001. Columns with different subscripts for physical and spiritual formidability differ at *P* < 0.05.

**Table 4.** Regression analysis of personal physical and spiritual formidability on willingness to fight and commit costly sacrifices for others (Studies 7 to 8)

Study	Predictor	*B*	*SE*	*t*	*P*	LLCI	ULCI
10	Physical formidability	−0.01	0.01	−0.76	0.448	−0.018	0.008
	Spiritual formidability	0.03	0.01	2.65	0.009	0.006	0.043
11	Physical formidability	0.68	0.43	1.57	0.117	−0.172	1.536
	Spiritual formidability	1.68	0.44	3.81	<0.001	0.811	2.549

LLCI, lower-level CI; ULCI, upper-level CI.

The results of Studies 10 and 11 replicated the previous studies by showing that spiritual formidability is more strongly correlated with costly sacrifices for the group than physical formidability. The USAFA cadet sample in Study 10 shows that spiritual formidability was positively correlated with costly sacrifices for the group and, notably, that the effect of spiritual formidability on costly sacrifices is mediated by group loyalty in predicting willingness to fight and sacrifice. The mediation relationship we found is not unique to USAFA cadets who have experienced military training. The relationship between spiritual formidability and loyalty on costly sacrifices generalized to European civilians, demonstrating the same relationship in an entirely different cultural population and setting, who may have other origins of group loyalty.

## Discussion

Although sometimes necessary or advantageous, participating in war is an extraordinarily costly endeavor causing loss, trauma, and a wide range of sacrifices to people for centuries. Why do humans resolve to persist in combat despite these costs? Previous studies have shown self-assessments of physical formidability, which are magnified in groups, inspire sacrifice in combat and other types of violence (3–9, 39). In the studies reported here from diverse populations, personal spiritual formidability, distinct from religiosity, consistently predicted costly sacrifices, whereas physical formidability did not. This pattern of results was found among general populations in different countries and a specially selected and trained population in which a willingness to fight and die is conceivable and even expected (i.e., USAFA cadets). Self-estimated spiritual formidability was rated higher than self-estimated physical formidability, and this bore out in expressed willingness to fight and die for others.

Like David when confronted with the threat of Goliath, a wide range of people are committed to engage in interpersonal and intergroup conflict because spiritual factors, whether secular or religious (10), are of greater importance than estimates of physical magnitude. Or, as Darwin put it in *The Descent of Man* (53), the virtues of “morality … patriotism, fidelity, obedience, courage, and sympathy,” as products of “natural selection,” could result, and has historically resulted, in low-power groups resisting and prevailing against materially stronger groups. He conjectured that groups populated by heroes and martyrs, better endowed with such virtues that “come to be highly esteemed or even held sacred,” would dominate in history’s unrelenting competition for survival.

Our findings critically extend previous research (12), showing that personal spiritual formidability is one of a number of elements driving the will to fight but one that is predominant in certain contexts and whose neglect may be critical to understanding failures of political and military strategy. Disentangling the summary representation of formidability into distinct physical and spiritual dimensions, we add more precision to the concept of formidability to better understand the contributing role of each in the will to fight. Given our findings that nonphysical elements are more critical to both materially inferior and materially superior groups, in conjunction with recent events in South Central Asia, this is a difference with a distinction.

Our results are consistent with the key tenet of the Formidability Representation Hypothesis (38, 54), namely that bodily dimensions are used by the mind to summarize diverse factors contributing to decision-making in situations of potential conflict. However, our work moves beyond prior investigations by demonstrating the value of separately querying participants regarding different factors that contribute to such decisions: by overtly contrasting material and nonmaterial components of formidability, we reveal the profound importance of the latter. In generalizing to a wider set of populations in the current studies, we found two distinguishable factors with a reliable relationship to the will to fight across a broad audience. Furthermore, the inner spiritual aspect of formidability relative to the physical aspect of formidability is a stronger predictor of the will to fight. This helps to distinguish the relative influence of the factors making up the concept of formidability in a significant way and creates the opportunity to study the relationship between these factors further. For example, one future direction for research could examine how physical and spiritual formidability may be correlated and determine the direction of causality: 1) spiritual formidability may increase as a result of an increase in physical formidability (being better equipped might make a combatant more motivated to fight), or 2) physical formidability may increase as a result of an increase in spiritual formidability (winning battles can increase one’s internal conviction and strength, which in turn can lead to more soldiers joining the cause, material support from allies, and demoralization or disbanding of the enemy’s army). It may be that the closer one gets to achieving a goal through physical sacrifice (e.g., as with the Taliban in 2021), perception of both the spiritual nature of the goal and the physical dimensions of sacrifice may become more closely joined and the correlation between spiritual and physical formidability consequently more pronounced.

Among all the groups studied in this effort, cadets are the most likely to risk their lives in combat. For them, personal spiritual strength is significantly influential in predicting the will to fight and sacrifice mediated by loyalty to an ingroup. The cadets preparing for military service revealed an individual predisposition (personal spiritual formidability) as a determinant of collective orientation (loyalty) leading to expressed decisions to fight and sacrifice for a primary ingroup. The mediated connection between individual character and group orientation (through loyalty) indicates that the connection is strong. For those in military training (e.g., cadets), the military tends to draw strong loyalty from its members (16), as individuality is systematically suppressed, and group goals and norms become paramount through early training experiences. The study of Spanish participants further suggests and confirms that spiritual formidability is more generally related to an increased willingness to sacrifice for groups because of loyalty. Taken together, loyalty appears to provide the link between an individual's tie to the spiritual sacred and the group to which they belong. Further investigation of how personal commitment can be related to collective commitment remains a key topic for understanding willingness to sacrifice. In addition to spiritual and physical formidability, aspects of sacred values, identity fusion, group cohesion, perceptions of resilience, social networks, and other factors likely will be variously involved (see refs. 48 and 55–57 for a pertinent review).

Most studies related to coalitional aggression, military planning, political science, and evolutionary psychology have considered physical formidability (along with leadership and cohesion) to be the critical factors in the willingness to fight. Few, however, have seriously considered, much less systematically investigated, the spiritual and value-driven dimensions of human conflict and their relationships with perceived formidability. As Fessler, Holbrook, and colleagues (12) have found, psychological dimensions associated with inner states of prospective combatants contribute to perceptions of relative formidability (11). Our contribution in this regard has been to extend these insights by teasing apart some of the physical and psychosocial factors involved in representations of formidability—factors suggested to us by actors and actions on the battlefield—and to reveal their relative strength in predicting expressions of costly sacrifice across different cultural groups. Even those studies that have examined religion and ideological commitment in this context consider spiritual strength only as a contributing factor, and not a primary or overriding factor, in decisions to fight or flee. Methodologically, our studies differed from previous studies in comparing self-perceptions of the dimensions within formidability assessments and how these factors influence decisions to fight and make sacrifices without assuming the primary role of physical capacities. Future studies are already underway to better understand why participants rate themselves as higher on spiritual relative to physical formidability and how these differences translate to willingness to sacrifice (e.g., for cause versus for comrade).

We expected broad differences between cultures: regional, military versus civilian, and materially superior versus inferior. Yet, the importance of inner strength of character was fundamental across populations, transcending cultural differences, training, manpower, and firepower. A paramount role for spiritual formidability in producing a willingness to fight, though empirically understudied, may seem somewhat unsurprising for religiously and ideologically motivated groups in the Middle East and elsewhere (51); however, in view of classical military doctrine and training, it is somewhat surprising for the US cadet population and for civilian populations and the military more generally.

These studies were designed to provide both a clearer “emic” (subjective and intentional) as well as “etic” (behavioral outcome-oriented) understanding of fundamental determinants that inspire expressed willingness to act in conflict—an understanding relevant to theories of motivation, aggression, warfare, and many other aspects of competitive and cooperative human behavior. Future studies are underway to examine these findings experimentally with behavioral and physiological (e.g., neural) measures to help bridge the chasm in social science research between what respondents say and what they do, along lines that we and colleagues have pursued for other significant determinants of the willingness to fight, such as sacred values (58), identity fusion (48, 49, 59), and physical formidability (60). Overall, our findings, in conjunction with recent events in the Middle East and Central Asia, suggest that failure to systematically assess the critical components of the nonmaterial, spiritual dimension of human conflict and willingness to sacrifice will continue to lead to strategic failures in political and military planning (61, 62). As General Mark Milley, the Chairman of the Joint Chiefs of Staff of the US military, noted in testimony to Congress on “the strategic failure” of the US political and military mission in Afghanistan, what he deemed “the intangible factor” of the will to fight was critical to the unanticipated outcome of America’s longest war: “We can count the trucks and guns and the units and all that. But we can’t measure a human heart from a machine” (63). Our aspiration for this line of research is to render tangible this spiritual dimension: to help us better understand the outcomes of past and ongoing conflicts as well as aid in predicting and hopefully preventing eruption of future conflicts.

## Materials and Methods

Data from 3,285 respondents in six different countries were used for the analysis. After receiving the Institutional Review Board (IRB) approvals identified in Table 5, participants from each country were recruited and contacted through a variety of methods, including face-to-face interviews, online social media, and flyers. Consents from every participant were obtained verbally using an IRB-approved script or signed via an informed consent document. The participants filled out surveys presented in the appropriate language of the country and asked to estimate their personal physical and spiritual formidability using the measures displayed in Fig. 1 (38, 64, 65) along with other ratings of interest. In Studies 1 to 3, we asked participants to rate their own religiosity to understand its relationship with our central constructs. In Studies 4 to 9, participants from Mosul, Iraq (*n* = 72, all males, age range 28 to 50), Casablanca, Morocco (*n* = 420, 49.5% females, age range 18 to 78), and Palestine (*n* = 730, 51.7% female, age range 18 to 85) used a tablet provided by researchers to complete the survey. Studies 4 to 8 used different methods to increase the generalizability of our findings. In Study 7, participants from Palestine (*n* = 470, 50.9% female, age range 18 to 77) completed a paper-and-pencil survey using items adapted from the previous studies. In Studies 8 and 9, participants from across Palestine (*n* = 171, 18.7% female, age range 18 to 71) and Lebanon (*n* = 321, 21.1% female, age range 18 to 65) completed their surveys online using technologies available to them. In Study 10, USAFA cadets (*n* = 120, 43.3% female, age range 17 to 25) completed the survey using a tablet similar to Studies 1 to 3 with an additional item for loyalty (*SI Appendix*). In Study 11, Spanish participants (*n* = 240, 64.6% female, age range 18 to 80) were recruited via Spanish online social media for an unpaid study. As in Studies 4 to 10, the participants were asked to estimate their perceived personal physical and spiritual formidability and the costly sacrifices they would be willing to make for their country. Loyalty to their country was assessed with a single item. Additional information on how loyalty and the will to fight were measured is in the *SI Appendix*.

**Table 5.** Sample characteristics, IRB information, and sample size determinations for Studies 1 through 11

Study	Source	*N*	Gender	Age (range)	IRB	Sample determination
1	Spain: Online study	202	114 F, 88 M	M = 39.0, SD = 13.7 (18–78)	National Distance Education University (UNED) Bioethics Committee No. 0720	No participants were excluded from the analyses
2	Spain: Online study	176	103 F, 72 M, 1 NA	M = 35.5, SD = 12.9 (18–72)	UNED Bioethics Committee No. 0720	No participants were excluded from the analyses
3	Spain: Online study	363	212 F, 151 M	M = 36.9, SD = 12.8 (18–72)	UNED Bioethics Committee No. 0720	No participants were excluded from the analyses
4	Iraq: Ethnographic field work immediately after the defeat of ISIS	72	0 F, 72 M	M = 39.5, SD = 9.1 (28–50)	ARTIS Research and Risk Modeling (RRM) IRB No. 2014-0925	No participants were excluded from the analyses
5	Morocco: Ethnographic field work in 2019 from neighborhoods linked to terrorist campaigns	420	209 F, 210 M, 1 NA	M = 34.7, SD = 12.74 (18–78)	ARTIS RRM IRB No. 2019-0329	No participants were excluded from the analyses
6	Palestine (a): Ethnographic field work in 2019 from neighborhoods linked to terrorist campaigns	730	377 F, 353 M	M = 43.3, SD = 15.37 (18–85)	ARTIS RRM IRB No. 2018-1214	No participants were excluded from the analyses
7	Palestine (b): Volunteer participants from around the country	470	239 F, 231 M	M = 38.2, SD = 13.2 (18–77)	ARTIS RRM IRB No. 2020-0407	No participants were excluded from the analyses
8	Palestine (c): Volunteer participants from around the country	171	32 F, 139 M	M = 30.5, SD = 11.1 (18–71)	ARTIS IRB RRM No. 2020-0407	No participants were excluded from the analyses
9	Lebanon: After the COVID outbreak, participants from across the country responded to social media ads	321	30 F, 115 M, 176 NA	M = 34.8, SD = 12.7 (18–65)	ARTIS IRB RRM No. 2020-0928	No participants were excluded from the analyses
10	United States: Surveys were completed in person by cadets enrolled at the Air Force Academy	120	52 F, 68 M	M = 20.3, SD = 1.7 (17–25)	USAFA IRB No. FAC20180020E	13 cadets did not complete aspects of the survey and excluded from the regression analysis
11	Spain: Participants from across the country	240	155 F, 85 M	M = 42.8, SD = 15.4 (18–80)	ARTIS RRM IRB No. 2018-0905	27 participants did not complete portions of the survey and were excluded from the regression analysis

F, female; M, male; NA, undisclosed.

## Supplementary Material

Supplementary File

## Data Availability

Anonymized human subjects data have been deposited in the Open Science Framework (https://osf.io/mvhgj/?view_only=10b8928478964e5684f8fa8ea7d3dbee) (66).
