# Supplementary material for: Spiritual over physical formidability determines willingness to fight and sacrifice through loyalty in cross-cultural populations
Source: Proc Natl Acad Sci U S A. 2022 Feb 7;119(6):e2113076119. doi: 10.1073/pnas.2113076119 (PMC8833214; doi:10.1073/pnas.2113076119)
Supplement: Supplementary File [file pnas.2113076119.sapp.pdf]

PNAS
www.pnas.org

Supplementary Information for
Spiritual over Physical Formidability Determines Willingness to Fight and Sacrifice through Loyalty in Cross-Cultural Populations

Chad C. Tossell[1], Angel Gómez[2,3], Ewart J. de Visser[1], Alexandra Vázquez[2,3], Bianca T. Donadio[1], Amanda Metcalfe[1], Charles Rogan[2], Richard Davis[2,4], Scott Atran[2,4,5]

[1]Warfighter Effectiveness Research Center (WERC), Department of Behavioral Sciences & Leadership, USAF Academy, CO 80840
[2]Artis International, 8325 W. Happy Valley Rd., Peoria, AZ 85383
[3]Departamento de Psicología Social y de las Organizaciones, Universidad Nacional de Educación a Distancia, UNED, C/ Juan del Rosal, No. 10, 28040 Madrid, Spain
[4]Changing Character of War Centre, University of Oxford, Mansfield Road, Oxford, OX1 3TD UK
[5]Gerald Ford School of Public Policy, University of Michigan, Ann Arbor, MI 48109

Corresponding Author: Chad Tossell, 2354 Fairchild Drive, USAF Academy, CO 80840
Email: chad.tossell@afacademy.af.edu

This PDF file includes:

Sensitivity Analyses for Sample Size Determination
Supplemental Methods and Measures
SI References

Sensitivity Analyses

In all studies, we performed a sensitivity analysis, using G*Power (1), to determine which would be the minimum size effect to reject the null hypothesis considering our sample size and assuming an alpha level of .05 and 80% power.

Study 1. The results indicate that an $p \geq .194$ for a correlation (point biserial model, two tails) would be enough to reject the null hypothesis.

Study 2. The results indicate that an $p \geq .208$ for a correlation (point biserial model, two tails) would be enough to reject the null hypothesis.

Study 3. The results indicate that an $\chi^2 \geq .3.841$ for a generic χ^2 test would be enough to reject the null hypothesis.

Study 4. The results indicate that an $f^2 \geq .140$ for a linear multiple regression (fixed model R^2 deviation from zero) would be enough to reject the null hypothesis.

Study 5. The results indicate that an $f^2 \geq .023$ for a linear multiple regression (fixed model R^2 deviation from zero) would be enough to reject the null hypothesis.

Study 6. The results indicate that an $f^2 \geq .013$ for a linear multiple regression (fixed model R^2 deviation from zero) would be enough to reject the null hypothesis.

Study 7. The results indicate that an $f^2 \geq .021$ for a linear multiple regression (fixed model R^2 deviation from zero) would be enough to reject the null hypothesis.

Study 8. The results indicate that an $f^2 \geq .057$ for a linear multiple regression (fixed model R^2 deviation from zero) would be enough to reject the null hypothesis.

Study 9. The results indicate that an $f^2 \geq .030$ for a linear multiple regression (fixed model R^2 deviation from zero) would be enough to reject the null hypothesis.

Study 10. The results indicate that an $f^2 \geq .082$ for a linear multiple regression (fixed model R^2 deviation from zero) would be enough to reject the null hypothesis.

Study 11. The results indicate that an $f^2 \geq .046$ for a linear multiple regression (fixed model R^2 deviation from zero) would be enough to reject the null hypothesis.

In Studies 1-3, all participants who completed all items assessing religiosity, spiritual formidability, and physical formidability were included in the analysis. Similarly, all participants who completed physical and spiritual formidability items and dependent measures in Studies 4-9 were included in the analysis. In Studies 10-11, all participants who completed these items were included in the replication analyses. Participants not completing the loyalty items were excluded from the mediation analysis. Table 5 in the main text contains a detailed description of each sample including Institutional Review Board (IRB) approvals.

Supplementary Methods and Measures

All of the survey items used in Studies 1-11 were validated in previous research (e.g., 1). Across all studies, physical and spiritual Formidability were always presented side by side on the same display regardless of the data collection method.

The measure of physical and spiritual formidability in English is as follows:

In the next set of questions you will see images that represent the physical and spiritual formidability of a person, group, country, or institution.

> *The physical formidability of a person or a group represents the ability and material resources (e.g., access to weapons, size, strength) of a person or group to fight and achieve their objectives. Physical formidability endows the person or group with the material potential to defend themselves or inflict physical damage to the opponent.*
>
> *The spiritual formidability of a person or group symbolizes the ability and immaterial resources (internal energy, perseveration, strength of belief) of a person or group to fight*

and achieve their goals. It refers to the force with which one believes for what ones struggles and defends.

The measure of physical and spiritual formidability in Arabic is as follow:

الجسدية والروحية/المعنوية لشخص أو مجموعة أو دولة أو مؤسسة.

تعبر القوة المادية أو الجسدية للشخص أو للمجموعات عن امتلاك القدرة والإمكانات المادية المتاحة (مثل القوة الاجتماعية وامتلاك الاسلحة وكثرة العدد وكبر الحجم) لخوض المعارك وتحقيق أهدافها. تمنح القوةُ المادية الفردَ أو الجماعة إمكانية الدفاع عن النفس وإيقاع الخسائر بالأعداء.

ترمز القوة الروحية أو المعنوية للشخص أو للجماعة إلى قدرات ومصادر غير مادية (مثل الطاقة الداخلية، قوة الإرادة، القدرة على الصمود، وقوة الإيمان) بحيث تمكنهم من خوض المعارك وتحقيق أهدافهم. القوة الروحية أو المعنوية تمنح الشخص أو الجماعة قوة الإرادة اللازمة لتحقيق أهدافهم والصمود ومواجهة التحديات والتغلب على العقبات. تشير هذه القوة الروحية إلى القوة التي يؤمن بها الفرد وإلى ما يقاتل من أجله ويدافع عنه.

The measure of physical and spiritual formidability in Spanish is as follow:

En el siguiente conjunto de preguntas usted encontrará una serie de imágenes que representan la percepción de la fuerza física y espiritual de una persona, grupo, país o institución

La percepción de la fuerza física de una persona o grupo representa la capacidad y los recursos materiales (por ejemplo, poder social, acceso a armas, tamaño, etc.) de una persona o grupo para luchar y lograr sus objetivos. La fuerza física confiere a la persona o grupo el potencial material para defenderse y/o infligir daño físico al oponente.

La percepción de la fuerza espiritual de una persona o grupo simboliza la capacidad y los recursos inmateriales (energía interna, perseverancia, fuerza en las propias creencias) de una persona o grupo para luchar y lograr sus objetivos. Se refiere a la fuerza con la que uno cree por lo que lucha y defiende".

Data for all studies are available on OSF at the following link:

https://osf.io/mvhgj/?view_only=10b8928478964e5684f8fa8ea7d3dbee

Studies 1-3

Religiosity was measured by a single-item scale asking participants to what extent they consider that most Spanish people are religious (from 0 = Not religious at all to 6 = Extremely religious) in Studies 1 and 2. In Study 3, we asked participants if spiritual formidability is more important to predict the behavior of a group than religiosity.

The religious and practicing group was not related to physical or spiritual formidability, $r(7) =.10$. $p=.79$ with physical and 0.01, $p = .98$ for spiritual. For those reporting they are religious but not practicing, religiosity was not related to spiritual formidability, r (70) = -.06, $p = .64$ and -.12, $p = .32$ and, for those reporting as non-religious, spiritual formidability and religiosity were not related, $r(93) = 0.05$. $p=0.66$ and 0.07, $p = .53$.

Studies 4-5

Physical and Spiritual Formidability ($\alpha_{avg} = .90$) - As described and displayed in the main text, physical and spiritual formidability were measured on a slider scale that enabled participants to increase or decrease the size and muscularity of an image of a male body. The smallest, thinnest

figure corresponded to a value of zero and the largest, most muscular figure corresponded to a value of one.

Willingness to Fight and Commit Costly Sacrifice (α_{avg} = .89) - Costly sacrifices for the country (i.e., ingroup) were measured by a five-item scale adapted from our previous study (1), on scales from 0 (totally disagree) to 6 (totally agree), where participants were asked to what extent, if necessary, they would be willing to display different kinds of self-sacrifice to defend their country as follow: “lose my job or source of income”, “go to jail”, “use violence”, “let my children suffer physical punishment”, and “die”. In Study 5, this scale includes a sixth item: "If necessary, I would be willing to be exiled from Morocco and be stripped from my Moroccans citizenship to defend Moroccans".

Studies 6-9

Physical and Spiritual Formidability (α_{avg} = .89) – Formidability measures mirrored Studies 1-2. Because Study 7 used a paper and pencil version of the survey, we used six bodies to measure each type of formidability (see Figure 1).

Willingness to Fight and Commit Costly Sacrifice (α_{avg} = .92) – This was measured similar to Studies 4-5 except Studies 6-9 did not include the item “let my children suffer physical punishment”. Studies 6 and 9 included two additional items: “physically suffer”, and “risk harm to friends close to me”.

Study 10-11

Physical and Spiritual Formidability – Measured via slider scales as described above for Studies 1-3 and 5-6.

Willingness to Fight and Commit Costly Sacrifice (α_{avg} = .92) – In Study 10, costly sacrifices for the country were measured by a five-item scale ranging from 0 (totally disagree) to 6 (totally agree), where participants were asked to what extent, if necessary, they would be willing to display different kinds of self-sacrifice to defend their country as follows: “lose my job or source of income”, “go to jail”, “use violence”, “let my children suffer physical punishment”, and “die”. Because cadets generally do not have children, the item “let my children suffer physical punishment” was replaced with “be a prisoner of war” in Study 10. In Study 11 the item “be a prisoner of war” was not included.

Loyalty – Group Loyalty was assessed by a single item asking participants how important it is for them to be loyal, or to show loyalty, to their group.

Mediation Analyses

The analyses used in Studies 10 and 11 to assess the extent to which loyalty to the group mediated the positive correlation between spiritual formidability and costly sacrifices controlled for physical formidability perceptions, age, and gender. For this mediation analysis, we utilized the bias-corrected bootstrapping procedure (5,000 samples) in the indirect macro for SPSS, model 4 (2).

SI References

1. E. Erdfelder, F. Faul, A. Buchner (1996). GPOWER: A general power analysis program. *Behavior Research Methods, Instruments & Computers,* **28(1)**, 1–11.
2. A. Gómez, S. Atran, et al., The devoted actor's will to fight and the spiritual dimension of human conflict. *Nat. Hum. Behav.* **1,** 673-679 (2017).
3. K.J. Preacher, A.F. Hayes , Asymptotic and resampling strategies for assessing and comparing indirect effects in multiple mediator models. *Behavior research methods,* **40***,* 879-891.
